# Duffy antigen receptor for chemokines gene polymorphisms and malaria in Mangaluru, India

**DOI:** 10.1186/s12936-019-2966-9

**Published:** 2019-09-24

**Authors:** Prabhanjan P. Gai, Welmoed van Loon, Konrad Siegert, Jakob Wedam, Suyamindra S. Kulkarni, Rashmi Rasalkar, Archith Boloor, Arun Kumar, Animesh Jain, Chakrapani Mahabala, Shantaram Baliga, Rajeshwari Devi, Damodara Shenoy, Pramod Gai, Frank P. Mockenhaupt

**Affiliations:** 1 Charité-Universitätsmedizin Berlin, corporate member of Freie Universität Berlin, Humboldt-Universität zu Berlin, and Berlin Institute of Health; Institute of Tropical Medicine and International Health, Berlin, Germany; 2 Karnataka Institute for DNA Research, Dharwad, Karnataka India; 3 Kasturba Medical College, Mangalore, India; 4 Manipal Academy of Higher Education, Manipal, Karnataka India; 5 District Vector Borne Disease Control Programme Office, Dakshina Kannada, Mangaluru, Karnataka India; 6 Wenlock Hospital, Mangaluru, Karnataka India

**Keywords:** Duffy, DARC, SNPs, Malaria, India, *Plasmodium vivax*, *Plasmodium falciparum*

## Abstract

**Background:**

Duffy blood group antigens serve as receptors for *Plasmodium vivax* invasion into erythrocytes, and they are determined by polymorphisms of the Duffy antigen receptor for chemokines (DARC), also known as Fy glycoprotein (FY). Duffy negativity, i.e., absence of the antigens, protects against *P. vivax* infection and is rare among non-African populations. However, data on *DARC* polymorphisms and their impact on *Plasmodium* infection in India are scarce.

**Methods:**

In a case–control study among 909 malaria patients and 909 healthy community controls in Mangaluru, southwestern India, *DARC* polymorphisms T-33C (rs2814778), G125A (rs12075), C265T (rs34599082), and G298A (rs13962) were genotyped. Associations of the polymorphisms with the odds of malaria, parasite species and manifestation were assessed.

**Results:**

Among patients, vivax malaria (70%) predominated over falciparum malaria (9%) and mixed species infections (21%). *DARC* T-33C was absent and C265T was rare (1%). FYB carriage (deduced from *DARC* G125A) was not associated with the risk of malaria per se but it protected against severe falciparum malaria (*P* = 0.03), and hospitalization (*P *= 0.006) due to falciparum malaria. Vice versa, carriage of *DARC* 298A was associated with increased odds of malaria (aOR, 1.46 (1.07–1.99), *P* = 0.015) and vivax malaria (aOR, 1.60 (1.14–2.22), *P* = 0.006) and with several reported symptoms and findings of the patients.

**Conclusion:**

This report from southern India is the first to show an independent effect of the *DARC* 298A polymorphism on the risk of malaria. Functional studies are required to understand the underlying mechanism. Moreover, FYB carriage appears to protect against severe falciparum malaria in southern India.

## Background

Malaria is considered a major driving force in shaping the human genome [1]. “Classical” erythrocyte variants such as the sickle-cell trait offer relative resistance against malaria and are thus subject to evolutionary selection in endemic regions. In addition, various further host genetic polymorphisms influence susceptibility to the disease and/or its manifestation [2, 3]. This includes the Duffy antigen receptor for chemokines (DARC, or Duffy antigen), which is a glycosylated erythrocyte membrane protein. The encoding *DARC* gene is located on chromosome 1. A common *DARC* polymorphism, G125A (rs12075), generates the FYA (G125) and FYB (125A) alleles. The resulting genotypes include the wildtype FYA/FYA, which correspond to the phenotype Fy (a+, b−), FYA/FYB (Fy (a+, b+)) and FYB/FYB (Fy (a−, b+)). An additional T-33C mutation silences antigen expression giving rise to Duffy blood group negativity (Fy (a−, b−)). Further single nucleotide polymorphisms (SNPs), C265T and G298A, are together responsible for weakening the expression of the FYB allele, whereas G298A alone is not able induce this effect [4].

In line with its function as a multi-specific receptor for a wide range of chemokines [5, 6], the absence of DARC on the erythrocyte cell surface (Duffy blood group negativity) has been associated with diverse conditions including inflammation, HIV infection, and malignancies [7, 8]. With respect to malaria, Duffy blood group negativity is predominant among Africans and renders erythrocytes resistant to invasion by *Plasmodium vivax* and *Plasmodium knowlesi* [5, 9–12]. Moreover, binding of DARC to platelet factor 4 (PF4) is essential for platelet-mediated killing of *Plasmodium falciparum* parasites [13, 14]. Associations of *DARC* genotypes with vivax malaria are reportedly conflicting. For instance in Brazil, FYA/FYA conferred reduced odds of vivax malaria [15]. However, in another Brazilian study, FYA/FYA was significantly more frequent in vivax malaria patients as compared to healthy blood donors without a history of malaria [11]. In India, the FYA allele has been associated with a reduced incidence of vivax malaria and the FYB allele with an increased one [16]. However, the few available individual studies from India did not show a link between *DARC* genotypes and vivax malaria [17–19].

In India, FYA/FYA is the predominant genotype and Duffy negativity occurs only in a few tribal populations [16, 19]. At the same time, India contributes to nearly half of the global *Plasmodium vivax* cases, and *P. vivax* and *P. falciparum* are responsible for 37% and 63% of malaria cases, respectively [20]. This provides the opportunity to study the effect of *DARC* genotypes on the risk of malaria per se, and of vivax and falciparum malaria separately. Of note, the manifestation of *Plasmodium* infection is not only caused by the infecting parasite but also by pro-inflammatory host responses, which potentially contribute to pathophysiology [21]. In this regard, the function of DARC as a receptor for diverse chemokines [5, 6] might possibly influence the clinical manifestation. Against this background, the present study aimed at describing the *DARC* genotype distribution pattern in Mangaluru and as a next step at examining the association of *DARC* genotypes with (i) malaria, (ii) malaria as caused by the various *Plasmodium* spp., and (iii) clinical presentation.

## Methods

A total of 909 malaria out-patients were recruited at Wenlock Hospital, Mangaluru, Karnataka, India between June to December 2015. Wenlock Hospital (900 beds) is the largest governmental hospital in Mangaluru offering treatment particularly for the economically-deprived part of the population. In parallel, an average of 40 (26–53) healthy community controls were randomly recruited in each of the 60 census wards of Mangaluru yielding a total number of 2478 individuals. The study protocol was approved by the Institutional Ethics Committee of Kasturba Medical College, Mangalore, Manipal University, and permission to conduct the study was granted by the Directorate of Health and Family Welfare Services, Government of Karnataka. Informed written consent was obtained from all individuals enrolled in this study.

Details of the patient recruitment process as well as socio-economic, clinical and laboratory data have been reported elsewhere [22]. Briefly, socio-economic data were collected by trained interviewers from patients (cases) and controls. Venous blood was collected into EDTA from malaria patients and by finger prick blood on Whatman™ 3MM paper from controls. Malaria parasites were counted *per* 200 white blood cells (WBCs) on Giemsa-stained thick blood films, and parasite species was defined based on thin-film microscopy. Following DNA extraction (QIAamp DNA Blood Mini kit, Qiagen, Hilden, Germany), *Plasmodium* species was ascertained by semi-nested polymerase chain reaction (PCR) assays [23]. Out of 2383 *Plasmodium*-negative controls, 909 were randomly selected for this case–control study. *DARC* SNP genotyping including T-33C, G125A, C265T and G298A was achieved by melting curve analysis on the Light Cycler 480 instrument (Roche, Basel, Switzerland) using commercial primers and probes; reagent concentrations and PCR conditions are available with the manufacturer (TIB MOLBIOL, Berlin, Germany).

Data analysis was performed using *R*Studio 3.5.1 (2018) (Integrated Development for R. RStudio, Inc., Boston, USA) and SPSS 25 (IBM Corp., Armonk, USA). The distribution of *DARC* genotypes between case and controls were compared by χ^2^ test or Fisher’s exact test as appropriate, and odds ratios (ORs) and 95% confidence intervals (95% CI) were calculated. Binomial logistic regression was used to calculate the adjusted odds ratios (aORs) of individuals with variant genotypes for malaria per se and for malaria separated by species with probable confounders: age, sex and migration to Mangaluru. Continuous parameters were compared using Student’s *t* test, Mann–Whitney U test, or Kruskal–Wallis test as applicable. A *P* value < 0.05 was considered statistically significant.

## Results

Essential characteristics of malaria patients and controls are displayed in Table 1. More than 90% of malaria patients were male adults. Their median age was 26 years, and more than three in four had migrated to Mangaluru a median period of 6 months before presentation (range 1–600 days). Their overall socio-economic status including educational background was low, and more than half of the patients were either construction workers or daily labourers [22]. In comparison, among control individuals, the proportion of males was lower, age was higher, and only a minority had migrated to Mangaluru city (each, *P* < 0.0005). Among patients, vivax malaria (70%) predominated over falciparum malaria (9%), and mixed *P. vivax*–*P. falciparum* infections (21%). The geometric mean parasite density (GMPD) was 3412/µl (95% CI 3081–3779).

**Table 1:** Characteristics of malaria patients and controls

Parameter	Cases	Controls	P
No.	909	909	
Male gender (%, n)	92.8 (844)	57.5 (523)	< 0.0001
Age (years; median, range)	26 (4–82)	30 (1–94)	0.0001
Migration (%, n)	77.8 (706/907)	34.4 (313/909)	< 0.0001
Socio-economic parameters			
No formal education (%, n)	33.0 (298/902)	11.1 (98/882)	< 0.0001
Occupation as construction worker or daily labourer (%)	56.1	36.3	< 0.0001
Monthly family income (rupees; median, range), cases; n = 893, controls; n = 575	6000 (0–35,000)	7000 (500–100,000)	0.06
Stated use of a bed net in preceding night (%, n)	39.1 (354,906)	54.3 (484/892)	< 0.0001
Stated use of a window net (%, n)	4.2 (38/906)	42.4 (376/890)	< 0.0001
Presence of stagnant water bodies (%, n)	31 (281/906)	3.3 (29/851)	< 0.0001
*Plasmodium* prevalence			
*P. vivax*	69.6 (633)	0	–
*P. falciparum*	9.0 (82)	0	–
*P. vivax* and *P. falciparum* mixed	21.3 (194)	0	–
Geometric mean parasite density (/µl; 95% CI)			
All patients	3412 (3081–3779)	–	–
*P. vivax*	2999 (2660–3382)	–	–
*P. falciparum*	5408 (3758–7750)	–	–
*P. vivax* and *P. falciparum* mixed	4246 (3413–5283)	–	–
*DARC* G125A genotypes (%)			
GG (FYA/FYA)	43.9	43.1	1
GA (FYA/FYB)	44.1	43.7	0.91
AA (FYB/FYB)	11.9	13.1	0.48
GA or AA (FYB carriers)	56.1	56.8	0.74
*DARC* G298A genotypes (%)			
GG	83.3	85.5	1
GA	15.6	13.3	0.16
AA	1.1	1.2	0.87
GA or AA	16.7	14.5	0.19

The *DARC* SNP-33 T>C was absent in 570 random samples genotyped, and the SNP 265 C>T was rare (1% (6/564) heterozygous). These polymorphisms were thus omitted from analysis. Genotyping of the *DARC* SNPs G125A and G298A was successful in all patients and controls. *DARC* 298A occurred exclusively when also 125A was present, i.e., on an FYB background (*P* < 0.0001). In the study sample and based on *DARC* G125A, FYA/FYB (43.9%) and FYA/FYA (43.5%) were the most common Duffy genotypes (FYB/FYB, 12.5%). Of note, these genotypes did not differ between cases and controls, and thus were not associated with the odds of malaria (Table 1), irrespective of stratification by parasite species (Table 2).

**Table 2:** Genotype distribution among malaria patients and controls separated by *Plasmodium* species

SNP	Genotype	Controls, n = 909	Malaria patients		
			*P. vivax*, *n* = 633	*P. falciparum*, *n* = 82	*P. vivax* and *P. falciparum, n* = 194
*DARC* G125A (n, %)	GG (FYA/FYA)	392 (43.1)	280 (44.2)	36 (43.9)	83 (42.7)
	GA (FYA/FYB)	398 (43.7)	277 (43.7)	34 (41.4)	90 (46.3)
	AA (FYB/FYB)	119 (13.1)	76 (12.0)	12 (14.6)	21 (10.8)
	GA or AA (FYB carriers)	517 (56.8)	353 (55.7)	46 (56.1)	111 (57.2)
*DARC* G298A (n, %)	GG	777 (85.5)	521 (82.3)	69 (84.2)	167 (86.1)
	GA	121 (13.3)	105 (16.6)	11 (13.4)	26 (13.4)
	AA	11 (1.2)	7 (1.1)	2 (2.4)	1 (0.5)
	GA or AA	132 (14.5)	112 (17.7)	13 (15.8)	27 (13.9)

**Fig. 1:**
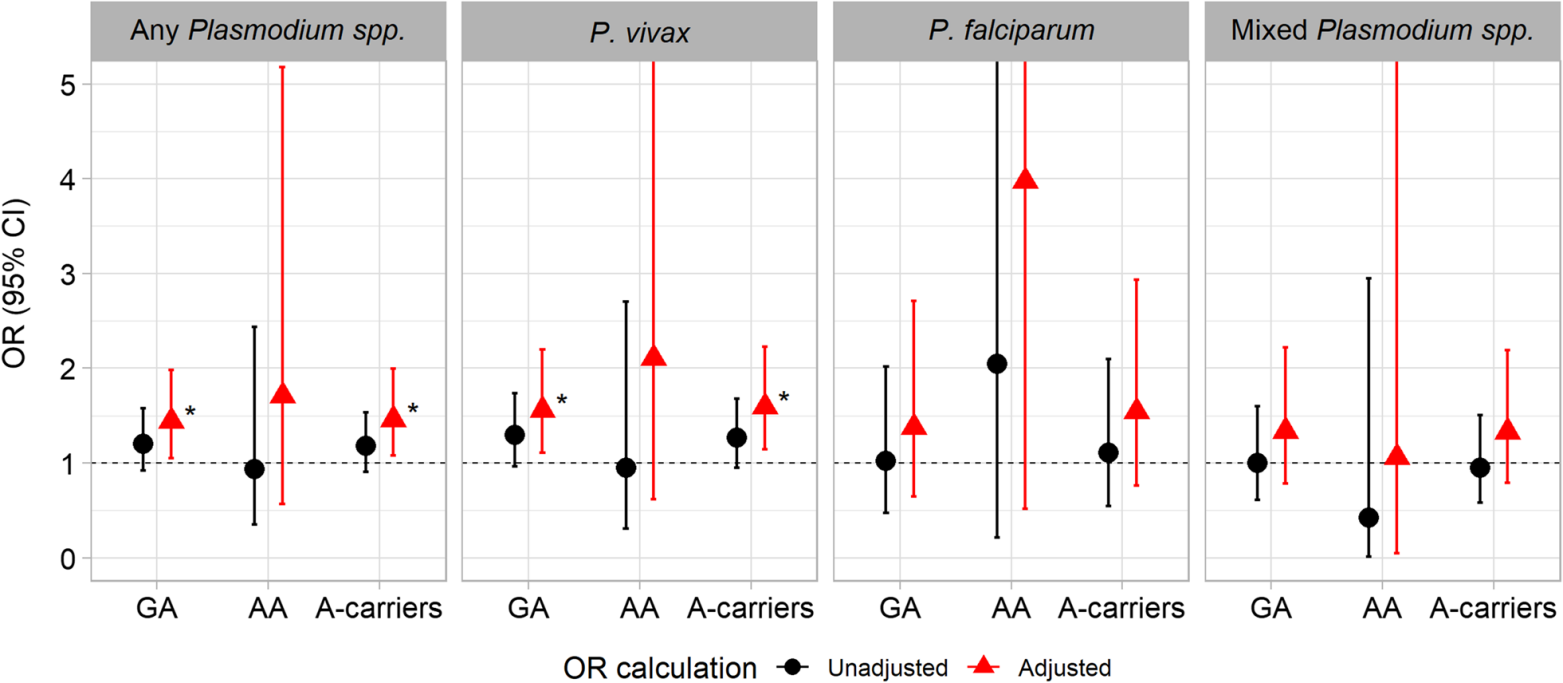
*DARC* 298A carriage is associated with increased odds of malaria and vivax malaria. OR, odds ratio; 95% CI, 95% confidence interval; unadjusted, crude odds ratio; adjusted, odds ratio adjusted for age, sex, and migration status; *P < 0.05 as compared to *DARC* G298 wildtype individuals. Forest plots display the odds of malaria according to *DARC* G298A genotypes. χ^2^ test or Fisher’s exact test was applied to calculate unadjusted odds ratios (95% CIs). Adjusted odds ratios were derived from logistic regression models adjusting for age, sex and migration status. As compared to control individuals (14.5%, 132/909), *DARC* 298A carriage was more common in malaria patients [16.7%, 152/909; aOR, 1.46 (1.07–1.99)] and in vivax malaria patients in particular [17.7%, 112/633; aOR, 1.60 (1.14–2.22)]

In contrast, carriage of *DARC* 298A (GA or AA), i.e., genotypes involved in but not solely responsible for a weakened expression of the FYB allele [4], appeared to be more common in malaria patients (16.7%, 152/909; *P* = 0.19) and in vivax malaria patients in particular (17.7%, 112/633; *P* = 0.09) as compared to controls (14.5%, 132/909) (Table 2; Fig. 1). Adjusting for the observed differences in age, sex, and migration, carriage of *DARC* 298A was associated with increased odds of malaria (aOR, 1.46 (1.07–1.99), *P* = 0.015) and of vivax malaria in particular (aOR, 1.60 (1.14–2.22), *P* = 0.006) (Fig. 1). No significant association with falciparum or mixed species malaria was observed.

In a next step of analysis among malaria patients, the proportions of hospitalization and of severe malaria were compared between *DARC* genotypes. For that, FYA/FYA and wildtype *DARC* G298, respectively, were set as reference groups. Among the patients, 3.5% (32/909) and 3.8% (35/909) of individuals were hospitalized and had severe malaria, respectively. The proportion of patients who were admitted to ward was highest in individuals with FYA/FYA (5.0%, 20/399), lower in FYA/FYB (3.5%, 14/401, *P* = 0.29) and lowest in FYB/FYB (0.9%, 1/109, *P* = 0.06). This was due to the absence of hospital admissions in patients with falciparum malaria carrying the FYB allele (*P* = 0.006, Table 3). Severe malaria due to any parasite species occurred at similar proportions in patients with the different FY genotypes, but severe falciparum malaria was absent in individuals carrying FYB (*P* = 0.03, Table 3). *DARC* 298A carriage did neither affect the proportion of hospitalized patients nor that of severe malaria. Also, it did not substantially change the associations of FYB carriage with the odds of hospitalization or severe malaria (Table 3).

**Table 3:** Proportion of patients with hospitalization and severe malaria according to *FY* genotypes

	FYA/FYA	FYA/FYB	FYB/FYB	FYB carriage with *DARC* 298A carriage
Hospitalization^a^				
All species	5.0% (20/399)	3.5% (14/401)	0.9% (1/109)	2.6% (4/152)
*P. vivax*	3.6% (10/280)	3.6% (10/277)	1.31% (1/76)	3.6% (4/112)
*P. falciparum*	16.7% (6/36)	0% (0/34)*	0% (0/12)	0% (0/13)
Mixed	4.8% (4/83)	4.4% (4/90)	0% (0/21)	0% (0/27)
Severe malaria^b^				
All species	3.5% (14/399)	3.7% (15/401)	2.8% (3/109)	3.3% (5/152)
*P. vivax*	2.9% (8/280)	3.6% (10/277)	2.6% (2/76)	3.6% (4/112)
*P. falciparum*	11.1% (4/36)	0% (0/34)	0% (0/12)	0% (0/13)
Mixed	2.4% (2/83)	5.6% (5/90)	4.8% (1/21)	3.7% (1/27)

^a^Of 35 patients admitted to ward, 10 were categorized as severe malaria patients. Other reasons included vomiting (5), dehydration (2), co-morbidities (2), weakness (2), suspected typhoid fever (1), jaundice (1), recent delivery (1), patient request (1), low blood pressure (1) as well as retrospectively not ascertainable causes (9)

^b^32 patients had severe malaria according to the WHO definition, i.e., hypotension (15; impaired perfusion not assessed), renal impairment (5), renal impairment and respiratory distress (1), severe malarial anaemia (4), prostration (3), confusion (2), jaundice (1), and abnormal bleeding (haematemesis, 1). Impaired consciousness, convulsions, hypoglycaemia, acidosis, hyperparasitaemia and pulmonary oedema were not observed

* *P* < 0.05 as compared to FYA/FYA

Lastly, signs and symptoms as well as laboratory parameters were analysed with respect to *DARC* genotypes. These did not differ significantly with the three FY genotypes. However, *DARC* 298A carriage was associated with increased proportions of patients reporting a history of muscle pain, back pain, fatigue, and at borderline, diarrhoea. Basically, the same findings were seen for vivax malaria, whereas in falciparum malaria *DARC* 298A carriage was associated with a history of sweats (*P* = 0.05) and of vomiting (Table 4). Clinically, the proportions of splenomegaly and of elevated bilirubin concentration were increased in patients with *DARC* 298A carriage as was axillary temperature (*P* = 0.05), specifically in mixed species infections (*P* = 0.01).

**Table 4:** Patient history as well as signs and symptoms according to *DARC* 298A carriage and parasite species

	All malaria patients		Vivax malaria		Falciparum malaria		Mixed species malaria	
	Wildtype	*DARC* 298A carriage	Wildtype	*DARC* 298A carriage	Wildtype	*DARC* 298A carriage	Wildtype	*DARC* 298A carriage
No	757	152	521	112	69	13	167	27
Reported signs and symptoms in preceding 2 weeks								
Fever	99.6 (753/756)	100.0 (152)	99.6 (518/520)	100.0 (112)	100.0 (69)	100.0 (13)	99.4 (166)	100.0 (27)
Headache	94.0 (711/756)	94.7 (144)	93.1 (484/520)	95.5 (107)	97.1 (67)	92.3 (12)	95.8 (160)	92.6 (25)
Muscle pain	84.9 (642/756)	91.4 (139)*	84.2 (438/520)	92.0 (103)*	89.9 (62)	84.6 (11)	85.0 (142)	92.6 (25)
Back pain	69.6 (526/756)	77.6 (118)*	67.9 (353/520)	77.7 (87)*	72.5 (50)	69.2 (9)	73.7 (123)	81.5 (22)
Fatigue/weakness	86.1 (651/756)	92.8 (141)*	85.0 (442/520)	91.1 (102)	89.9 (62)	92.3 (12)	88.0 (147)	100.0 (27)
Chills/shivering	88.1 (666/756)	90.1 (137)	88.1 (458/520)	90.2 (101)	82.6 (57)	84.6 (11)	90.4 (151)	92.6 (25)
Sweats	73.5 (554/754)	77.6 (118)	76.1 (395/519)	73.2 (82)	65.2 (45)	92.3 (12)*	68.7 (114/166)	88.9 (24)*
Cough	42.2 (319/756)	45.4 (69)	41.9 (218/520)	47.3 (53)	43.5 (30)	46.2 (6)	42.5 (71)	37.0 (10)
Nausea	41.2 (311/754)	44.1 (67)	40.8 (212/519)	42.0 (47)	42.0 (29)	38.5 (5)	42.2 (70/166)	55.6 (15)
Vomiting	29.8 (225/756)	29.6 (45)	28.5 (148/520)	22.3 (25)	30.4 (21)	69.2 (9)*	33.5 (56)	40.7 (11)
Diarrhoea	3.3 (25/756)	6.6 (10)	2.9 (15/520)	8.9 (10)*	5.8 (4)	0.0 (0)	3.6 (6)	0.0 (0)
Abdominal pain	31.7 (240/756)	29.6 (45)	30.0 (156/520)	32.1 (36)	40.6 (28)	23.1 (3)	33.5 (56)	22.2 (6)
Assessed parameters								
GMPD (/µl; 95% CI)	3364	3641	2995	3036	4681	11,580	4216	4428
	(3011–3757)	(2800–4734)	(2626–3415)	(2253–4092)	(3148–6961)	(5055–26,527)	(3340–5321)	(2347–8354)
Splenomegaly (%, n)	16.8 (126/751)	27.0 (41)*	14.5 (75/516)	25.9 (29)*	27.9 (19/68)	38.5 (5)	19.2 (32)	25.9 (7)
Axillary temperature (°C, mean ± SD), n = 903	37.2 ± 1.6	37.4 ± 1.5	37.1 ± 1.6	37.3 ± 1.4	37.3 ± 1.7	37.4 ± 2.0	37.3 ± 1.5	38.1 ± 1.4*
Body mass index (BMI) (kg/m^2^; median, range), n = 887	19.5 (12.2–46.3)	19.3 (13.6–33.3)	19.8 (12.3–39.5)	19.3 (13.6–33.3)	19.1 (12.6–27.5)	19.7 (14.6–24.9)	19.0 (12.2–46.3)	19.3 (16.7–31.9)
Hypotension (systolic BP < 80 mm Hg; %, n)	1.6 (12/756)	2.0 (3)	1.0 (5/520)	1.8 (2)	1.4 (1)	0.0 (0)	3.6 (6)	3.7 (1)
Severe thrombocytopenia (< 50,000/µl; %, n)	12.3 (88/715)	12.5 (18/144)	10.5 (52/495)	13.0 (14/108)	21.0 (13/62)	9.1 (1/11)	14.6 (23/158)	12.0 (3/25)
Anaemia (%, n)	34.3 (248/722)	35.4 (52/147)	30.1 (151/501)	33.9 (37/109)	41.0 (25/61)	41.7 (5/12)	45.0 (72/160)	38.5 (10/26)
Increased creatinine (> 1.4 mg/dl; %, n)	3.3 (23/688)	1.4 (2/140)	2.7 (13/473)	1.0 (1/103)	4.9 (3/61)	8.3 (1/12)	4.5 (7/154)	0.0 (0/25)
Increased bilirubin (> 1.2 mg/dl; %, n)	46.8 (324/693)	56.1 (78/139)*	46.3 (221/477)	57.3 (59/103)*	53.3 (32/60)	50.0 (6/12)	45.5 (71/156)	54.2 (13/24)

* *P* < 0.05 as compared to the wildtype *DARC* G298

## Discussion

The present results indicate that FYB carriage in an Indian population does not influence the risk of malaria per se, but, in case of *P. falciparum* infection, it is associated with protection from hospitalization and severe malaria. Vice versa, *DARC* 298A carriage appeared to increase the risk of malaria, and of vivax malaria in particular, and to affect the occurrence of several symptoms.

Despite its sample size the study has several limitations which need to be considered when interpreting the results: subgroups, e.g., patients with falciparum malaria, were relatively small affecting the power of analyses. Patients and controls differed in essential parameters such as age, gender, and migration status, because of which risk estimates had to be adjusted accordingly. The *DARC* polymorphisms T-33C and C265T were too rare to deduce meaningful findings. No interaction in terms of associations with malaria or signs or symptoms was seen for the FYA or FYB alleles and *DARC* G298A. Therefore, data were presented separately.

In the present study, the FYA/FYA and FYA/FYB genotypes occurred in each approximately 44%. Among more than 3000 blood donors in New Delhi, FYA/FYA and FYA/FYB were observed in 32.5% and 48.9%, respectively. Duffy blood group negativity, absent in the present study, was observed in 0.3% [24]. Of note, the proportion of Duffy blood group genotypes differs across India but findings from the South of the country closely match with the prevalence data of the present study [16]. In comparison to other ethnic groups, the predominant FYA/FYB genotype in the present study is slightly more common in Caucasians (49%) but rare in sub-Saharan Africans (1.0%) and Chinese (8.9%). This is due to a higher FYA allele frequency among Indians than in Caucasians and sub-Saharan Africans. Duffy blood group negativity is absent or very rare in all populations except for sub-Saharan Africans (68%) [24]. Carriage of *DARC* 298A was found in 15.6% of the current study participants, corresponding to an allele frequency of 0.08. Based on the 1000 Genomes Project, this matches the respective figure of 0.09 among South Asians, but it is lower than the allele frequency of 0.18 among Caucasians and higher than the value of 0.005 in Africans [4].

Duffy blood group antigens are known to play an important role in *P. vivax* malaria [5, 6, 25] and to be essential in platelet-mediated killing of *P. falciparum* [13]. However, actual findings in various populations including Indians have been ambiguous [11, 15–19]. In the present study FYA or FYB did not affect the odds of malaria, irrespective of parasite species.

This contrasts with recent report on protective effects of FYA/FYA against vivax malaria in Brazil [15]. Likewise, in one study from India, FYA was found to be associated with a reduced 5 years average incidence of vivax malaria [16]. In Brazil, no association with falciparum malaria was observed [15], in India, falciparum malaria was not analysed [16]. In another study from Brazil, FYA/FYA was associated with increased susceptibility to vivax malaria [11], and in older work from India, no impact of the Duffy blood group genotypes on vivax or falciparum malaria was observed [17–19]. The reason for these conflicting results may be related to variable proportions of *P. vivax* and *P. falciparum* among the patients included, partially low sample sizes and genetic variation among the diverse populations, including the Indian one. Of note, in the present study, falciparum malaria patients with FYA/FYA showed the highest rate of hospitalization and severe malaria, which was unexpected considering the protective effects against vivax malaria mentioned above [15, 16]. In-vitro, binding of Duffy antigens to platelet factor 4 (PF4) is crucial for platelet-mediated killing of *P. falciparum* [13, 14] even though the role of FY variation in that is unknown. One explanation for the finding of reduced odds of severe malaria in patients with FYB carriage could be that it affects binding affinity towards PF4 and thereby the capacity of platelet mediated killing. On the other hand, parasite densities and other severity markers of infection were not reduced in patients with FYB carriage. Consequently, further work is required to explain the observed association of FYB carriage with hospitalization and severe falciparum malaria.

A novel finding is that carriage of the *DARC* 298A variant increased the odds of malaria by roughly 50%. Moreover, this polymorphism was associated with increased proportions of patients reporting several signs and symptoms. This SNP has not been observed to be independently associated with malaria. One previous study from Brazil did not observe an association with malaria susceptibility when combining *DARC* C265T and G298A as a condition weakening the expression of Duffy antigens (FYX) [11]. *DARC* C265T was absent in the present study population. *DARC* 298 G>A results in an amino acid substitution in the first intracellular loop of the Duffy glycoprotein. It has been linked with reduced FYB expression only in the presence of C265T [4, 26]. On the other hand, DARC acts as a multi-specific receptor for chemokines. These include the melanoma growth stimulatory activity, interleukin-8, regulated upon activation normal T-expressed, monocyte chemotactic protein-1, neutrophil activating protein 2 and 3, epithelial neutrophil activating peptide-78, angiogenesis-related platelet factor 1, and growth-related gene alpha [5, 6]. In line with this wide-range receptor function, DARC per se has been associated with several inflammatory and infectious diseases including increased rates of prostate cancer and asthma as well as an increased risk of HIV infection in its absence [7, 27]. DARC also influences inflammation in terms of chemokine levels and leukocyte trafficking and malignancy [8]. Monocytes and neutrophils phagocytize infected red blood cells, and they are important sources of cytokines, which act as signaling molecules in activating immune responses against malaria [28]. Increased phagocytic activity via neutrophils is observed in vivax malaria [29]. A possible explanation in support of the present study findings could be the involvement of variant *DARC* 298A in altering the chemoattractant properties of the Duffy glycoprotein, leading to a modified activation of the pro-inflammatory signal cascade. Functional studies are needed to verify this hypothesis.

## Conclusion

This study from southern India is the first to show an independent effect of *DARC* 298A in *Plasmodium* infection. *DARC* 298A carriage appears to be associated with increased susceptibility to malaria and to vivax malaria in particular, and to worsen several signs and symptoms. Functional studies on the role of this polymorphism are required to disentangle the underlying mechanisms. The same applies to the role of FYB genotype carriage protecting against severe falciparum malaria. Considering Duffy blood group antigens being studied as vaccine candidates against vivax malaria [30] and the present clinico-epidemiological findings, unravelling the molecular mechanisms of Duffy blood group antigens influencing malaria susceptibility and resistance is urgently needed.

## Data Availability

The dataset generated and/or analysed in this study is not publicly available due to issues of confidentiality and ongoing analyses, but are available from the corresponding author on reasonable request.

## Abbreviations

DARC: Duffy antigen receptor for chemokines
FY: Fy glycoprotein
*P. vivax*: *Plasmodium vivax*
*P. falciparum*: *Plasmodium falciparum*
WBCs: white blood cells
PF4: platelet factor 4
SNP: single nucleotide polymorphism
PCR: polymerase chain reaction
ORs: odds ratios
CI: confidence interval
GMPD: geometric mean parasite density
BMI: body mass index
